# Flexible Composites with Variable Conductivity and Memory of Deformation Obtained by Polymerization of Polyaniline in PVA Hydrogel

**DOI:** 10.3390/polym14214638

**Published:** 2022-10-31

**Authors:** Andrei Honciuc, Ana-Maria Solonaru, Mirela Teodorescu

**Affiliations:** “Petru Poni” Institute of Macromolecular Chemistry, Aleea Gr. Ghica Voda 41A, 700487 Iasi, Romania

**Keywords:** conductive rubber, conductive polymers, conductive composites, polyaniline, hydrogel, viscoelastic composites

## Abstract

Flexible materials that provide an electric, magnetic, or optic response upon deformation or tactile pressure could be important for the development of smart monitors, intelligent textiles, or in the development of robotic skins. In this work we demonstrate the capabilities of a flexible and electrically conductive polymer material that produces an electrical response with any deformation, namely the electrical resistance of the material changes proportionally with the deformation pressure. Furthermore, the material exhibits a memory effect. When compressed beyond the elastic regime, it retains the memory of the plastic deformation by increasing its resistance. The material was obtained by in situ polymerization of semiconducting polyaniline (PANi) in a polyvinyl alcohol/glycerol (PVA/Gly) hydrogel matrix at −17 °C. Upon drying of the hydrogel, an elastomer composite is obtained, with rubber-like characteristics. When compressed/decompressed, the electrical resistance of the material exhibits an unusually long equilibration/relaxation time, proportional with the load applied. These phenomena indicate a complex relaxation and reconfiguration process of the PANi/PVA elastomer matrix, with the shape change of the material due to mechanical stress.

## 1. Introduction

The rapid developments in electronic monitoring devices, especially those with medical purpose, require the development of advanced materials acting as an interface between the electronic device and the human body [1]. Among these, flexible and wearable materials that are capable of translating human motion in electrical signal changes could be of valuable importance in development of corrective motion therapies, monitoring of the recovery of trauma patients, development of interactive prosthetics, but also in novel motion biometrics for enhanced security and identification [2,3]. The ideal candidates for such applications are flexible elastomeric materials, which have conductive properties and could generate a response to mechanical stress. Several types of strategies for obtaining elastomer materials with conductive properties have been previously reviewed [4]. 

In this work, we introduce a facile method for obtaining a viscoelastic composite with conductive properties obtained by in situ polymerization of polyaniline (PANi) semiconductor in a polyvinyl alcohol/glycerol (PVA/Gly) hydrogel matrix. The hydrogel matrix is allowed to dry and the PANi/PVA hydrogel becomes a rubber-like material, that is flexible and compressible, with conductive properties. The PANi is a well-known semiconducting polymer [5,6,7,8], has a high electrical conductivity [9,10,11,12], on the order of 10 S/cm, depending on the preparation method in the presence of various organic or inorganic acids, has a stable redox activity, and environmental stability [13,14,15]. One of the major known drawbacks of polyaniline is its poor processability in solvents and especially in aqueous solvents [16]. The key element in the production of a homogeneous polymer composite between insoluble PANi and a PVA hydrogel matrix, a quintessentially hydrophilic system, is the polymerization of PANi in a gelified, viscous stage of the PVA/Gly hydrogel. The PVA hydrogel matrix is a biocompatible material that can be molded in various shapes and forms, including thin films, membranes, and various blocks [17,18]. PANi/PVA hydrogels have been prepared in various ways in the past decade and various preparation recipes have been reported [19]. Hu et al. [20] have prepared PANi/PVA hydrogel by mixing a previously prepared PANi hydrogel and a PVA hydrogel; the result was a stretchable PANi/PVA composite that was used in the construction of flexible supercapacitor. Li et al. [21] prepared PANi/PVA hydrogel by the freeze thawing method, whereas the polymerization of PANi was conducted in the presence of PVA at −20 °C. They found that the PANi greatly improved the mechanical properties of the PVA hydrogel, conferring on it an 18.7-times higher compressive strength than that of pure PVA hydrogel. Further, Stejskal et al. [22] have prepared PANi/PVA cryogels, by simply mixing aniline hydrochloride with ammonium peroxydisulfate in aqueous solution of 5 wt% PVA (MW 61,000), whereas the liquid mixture was sucked into a syringe and quickly frozen with a solid CO_2_/ethanol suspension and left in a freezer at −25 °C for 5 days. Because of the cryogenic method used, the PANi grew in a microporous network surrounding the ice crystals. The electrical conductivity of the obtained cryogel was ca. 10^−3^ S/cm. While most reports have focused on electrical and mechanical studies and analysis of the PANi/PVA hydrogels as obtained, immediately after preparation, no investigations performed on the PANi/PVA composite resulted after natural drying of the material. Since hydrogels are largely aqueous, their electrical conductivity will be mostly ionic, and the intrinsic electronic conductivity of polymers will contribute to a lesser extent. In contrast, in this work we focus on investigating the electrical and mechanical properties of the PANi/PVA viscoelastic composite resulting after a minimum of 36 days of drying the hydrogel, which was prepared via the cryogenic method. In doing so, we aimed to obtain a material that is stable in time, most of the water has been removed, and its electrical conductivity is not purely due to presence of water but the intrinsic PANi conductivity plays a major role in the observed electrical properties of the material with the applied mechanical stimuli. To obtain the optimum viscoelastic composite, several PANi/PVA hydrogel compositions were prepared via the cryogenic method while varying the relative ratio of PANi/PVA and Gly/PVA in the composition. We observed that while the addition of PANi hardens the material, the elastic properties could be tuned by the addition of Gly in the polymer matrix, which plays the role of both cross-linker and plasticizer [23]. Upon drying the material, after the completion of the reaction, the elastomer exhibits a proportional response in the resistivity with compression, which makes it suitable for use in pressure sensors, tactile sensors, walking sensors, wearable textiles and a plethora of other applications that require conversion of mechanical pressure into a readable electrical signal. Such materials could be useful in developing “artificial skins” for humanoid robots, which could easily interpret and process the tactile pressure into electrical signals, as a means for improved sensorial interaction with their environment [4,24,25]. 

Combining a hydrogel and a conductive polymer has been a strategy to obtain multifunctional materials, and these were extensively reviewed [23,26,27]. While the addition of an electrically insulating component is expected to decrease the electrical conductivity, the gain in the material’s mechanical properties appears to compensate for this and produce an overall benefit. Single-component hydrogels based on conductive polymers have been reported in literature: for example, poly(3-thiopheneacetic acid) hydrogels were fabricated by covalently cross-linking the polymer with 1,1′-carbonyldiimidazole (CDI) in DMSO followed by solvent exchange [28]; in addition, a poly(4-(2,3-dihydrothieno[3,4-b] [1,4]dioxin-2-yl)methoxybutane 1-sulfonate) has been prepared from the corresponding water soluble monomer and cross-linked with a Cr^3+^ ion [29]. Single-component PANi hydrogels obtained by cross-linking the conductive polymer with phytic acid has also been reported [30]. However, the single-component hydrogels have the advantage of high conductivities, being free of an electrically insulating matrix, but exhibit elasticity only in the wet hydrogel state. Upon drying they become brittle and hard materials. Although a super-elastic polypyrrole hydrogel has been reported in literature [31], the mechanical properties of the material after dehydration of the hydrogel have not been reported. In this work, we focus on the PANi/PVA composite, after drying the hydrogel, and when the material is in its stable state. The presence of Gly in the composite confers the material with permanent flexibility and elasticity that does not change over time.

## 2. Experimental Methods

### 2.1. Materials

Poly(vinyl alcohol) (PVA) granules with an average molecular weight (Mw) of 12.4 × 10^4^ g/mol and a 99–100% degree of hydrolysis and Glycerol (Gly) (99.6%) were purchased from Acros Organics (Geel, Belgium). Aniline (>98%) was purchased from TCI Europe N.V. (Zwijndrecht, Belgium), ammonium persulfate (APS) (>99%) from Sigma Aldrich (Merck, KGaA, Darmstadt, Germany), hydrochloric acid (HCl) (≥37%) from Fluka (Honeywell Specialty Chemicals, Seelze, Germany). All the aqueous solutions were prepared in freshly distilled water.

### 2.2. Synthesis of PVA/Gly Hydrogel

The PVA hydrogels were obtained from polymeric solutions of 5% wt concentrations, by first dissolving the appropriate amount of PVA in distilled water and stirred vigorously for 3 h, at 1500 rpm and 90 °C, until the solution was homogeneous. Then, different mixtures with Gly were prepared with the following PVA/Gly weight ratios: 1:0, 1:2, 1:5, and 1:10 respectively. Each PVA/Gly mixture was split in two parts: one part was poured into clean and dried flasks and subjected to freezing at −17 °C, for 24 h, and then left to dry at room temperature, and the other part was further used for the synthesis of PANi/PVA composites.

### 2.3. Synthesis of PANi/PVA Composite

For PANi/PVA composite synthesis, ANi was first added dropwise into each mixture of PVA/Gly solutions with different weight ratios, maintaining a constant stirring of 700 rpm, at room temperature, until a homogeneous mixture was obtained. The weight ratio of ANi/PVA was 0:1, 1:1, 2:1, and 5:1, respectively. Then, APS initiator was dissolved in HCl 1M and also added dropwise to the homogenous mixture (molar ratio ANi/APS was 1:1). In the end, the mixtures were acidified with a concentrated HCl solution of 37% (ratio ANi/HCl was 5:1 *v*/*v*). The final solutions were poured into molds and frozen at −17 °C, for 24 h. The PANi/PVA hydrogels thus obtained are left to dry for 36 days until their shape remains stable.

### 2.4. Electrical Measurements

Electrical measurements of the PANi/PVA composites were carried with a custom-made set-up. To determine the conductivity of the composite as a function of composition, current–voltage (I–V) measurements were performed on various samples having a thickness ranging between 0.1–4.6 mm. The measurement was performed in a four-point probe configuration with disk-shaped copper electrodes, see Figure S1 in Supporting Information (SI), whereas the four-point contacts were achieved by using hemispherical copper disks glued together by electrically insulating epoxy resin. I–V measurements were acquired by applying a current ramp with a Keithley current source (Keithley 6220) and the voltage was read with a Keithley nanovoltmeter (Keithley 2127). The resistance of the sample with compression was determined on cylindrical PANi/PVA composites by attaching the top electrode to a micromanipulator (World Precision Instruments GmbH, Berlin, Germany, Micro Manipulator M3301R 3 Axis, 0.01 mm precision). The compression was carried from the top by moving the top electrode in the downward direction. The compression of the sample was performed in steps of 0.25 mm or 0.5 mm and the resistance was monitored live by performing I–V measurements with the same current source and voltmeter at a sampling rate of approximately one I–V measurement per second, while resistance or conductivity was automatically calculated from the I–V curves.

### 2.5. Mechanical Measurements

Mechanical measurements were performed on cylindrical PANi/PVA composites samples using the same moving electrode configuration as described above. The strain–stress curves were generated by compressing the cylindrical sample on its long axis by moving the top copper plate electrode downwards in steps of 0.25 mm. The force was measured by a technical weighing scale and the weight registered at each compression step was converted to the stress (force/area) applied. Simultaneously, at each compression step, the diameter of the cylinder was measured with a caliper tool (0.01 mm precision).

### 2.6. Material Characterisation

Scanning electron microscopy was performed with a Verios G4 UC Scanning Electron Microscope (SEM) from Thermo Fischer Scientific (Eindhoven, The Netherlands) using an Everhart–Thornley detector (ETD) and a beam energy between 4.5–5.4 eV. FTIR spectra were recorded by ATR using a DIGILAB-FTS 2000 spectrometer (Bruker, Germany) in the range of 400 to 4000 cm^−1^.

## 3. Results and Discussions

### 3.1. Preparation of the PANi/PVA Composite

**Figure 1:**
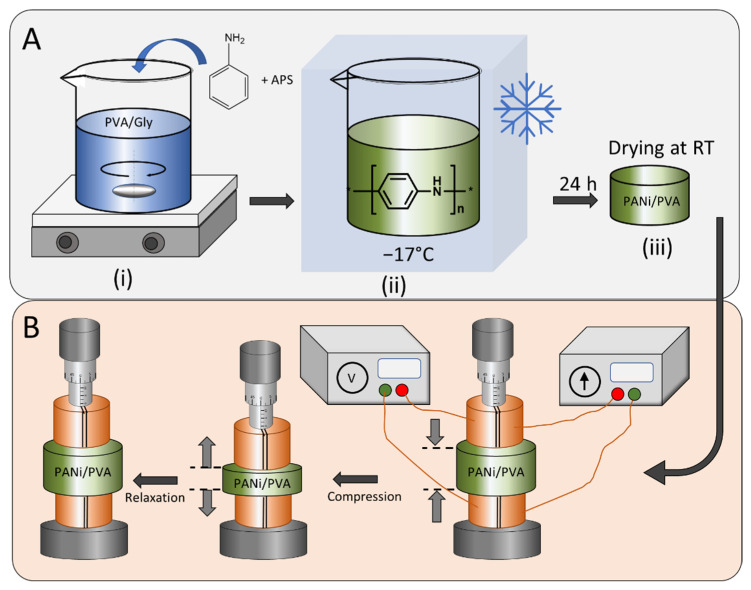
(**A**) Preparation procedure of the PANi/PVA elastic composite starting from (i) the preparation of the hydrogel matrix as reaction medium for the aniline polymerization, followed by the addition of the aniline and the polymerization initiator APS, (ii) cooling of the mixture and the polymerization initiation of PANi, (iii) removal of the sample from the cooling chamber and drying at room temperature. (**B**) The resistance and conductivity of the elastic PANi/PVA composite was measured with compression and relaxation cycles in a four-point electrode configuration and the mechanical compression was controlled by a micromanipulator.

The flexible PANi/PVA material was obtained by in situ polymerization of PANi semiconductor in a PVA/Gly hydrogel matrix. The synthesis steps are depicted in Figure 1, which illustrates the preparation of the PVA/Gly matrix, where the PVA (Mw = 12.4 × 10^4^ g/mol) was first dissolved in distilled water followed by addition of Gly, as described in the experimental methods. Upon mixing and heating at 90 °C, for 3 h, a homogeneous hydrogel was obtained. Before cooling, the ANi monomer was added together with the polymerization initiator APS. The polymerization reaction of ANi is exothermic and the polymerization reaction was initiated by cooling the reaction mixture at −17 °C in a freezer. After 24 h the reaction vessel was removed from the freezer and allowed to dry. Adjustment of the relative concentrations of the components yields different material properties, see Table 1 and Table S1. All PANi/PVA hydrogels obtained were allowed to dry at room temperature and ambient conditions for ca. 36 days. The material properties were inspected, and several observations can be made, see Table S1. In the absence of PANi and Gly, the PVA is hard, glassy, and brittle (sample AM3 in Table S1 and Table 1); the SEM image of AM3 in Figure S2B shows a featureless material surface. Addition of Gly in a proportion of PVA/Gly 1:1 (g/g) renders the material soft and sticky (sample AM4 in Table S1); the SEM image of AM4 in Figure S2C shows a non-porous, slightly fibrous material surface. In the absence of Gly, the addition of PANi in a proportion of PANi/PVA 1:1 (g/g) yields a non-flexible, hard rubbery material (sample AM1 in Table S1 and Table 1); the SEM image of AM1 in Figure S2A shows a fibrous and microporous material surface. Use of both components PANi/PVA and PVA/Gly in a proportion of 1:1 and 1:2 (or 1:5) respectively, leads to a soft, rubber-like material with good elasticity and compressibility (sample AM7 in Table S1 and Table 1); the SEM image of AM7 in Figure S2D shows a fibrous and microporous material surface. Any other composition, when PANi/PVA is used at more than a 1:1 ratio, yields a brittle rubber material, regardless of the concentration of Gly. The composition influences the sample’s elasticity and softness because the nature of the three components is different. Namely, PANi has a rigid molecular chain and typically, an increasing amount of PANI in a polymer blend leads to a reduction of the elongation at break [32]. PVA has excellent film-forming properties, high bonding, and antistatic properties and the Gly is a plasticizer, which improves the flexibility and brittleness of films. In fact, Gly is a well-known plasticizer used to improve the mechanical properties of biopolymers, such as starch [33], chitosan [34], whey proteins [35], etc.

**Figure 2:**
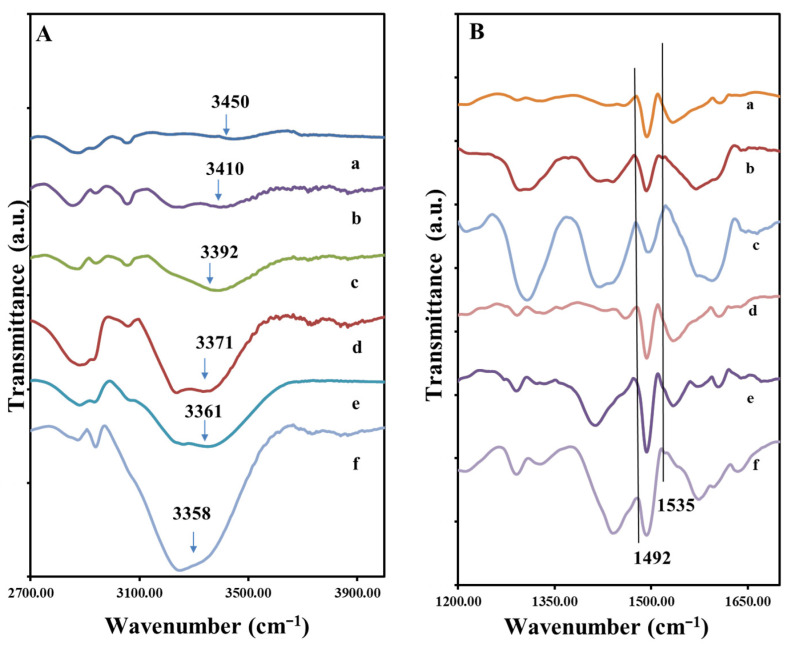
FTIR spectra of different composition of hydrogels (**A**) from top to bottom in the order of increasing Gly concentration in the composites (a) AM2, (b) AM10, (c) AM9, (d) AM7, (e) AM11, (f) AM8; (**B**) spectra of composite organized from top to bottom in the order of increasing PANi (a) AM2, (b) AM7, (c) AM8, (d) AM9, (e) AM10, (f) AM11.

The FTIR spectra of PVA, Gly and PANi, as well as that of composites AM2, AM7, AM8, AM9, AM10 & AM11, are given in Figure S3 and Figure 2.

Figure 2A presents the FTIR spectra of the samples which contain all three components (PVA, PANi and Gly), but in different weight ratios, see Table 1. It can be observed that almost all bands specific to the individual component appear, with some changes depending on their concentration in the sample. The biggest modifications are given by the band belonging to the hydroxyl group of PVA and Gly. In the samples with high content of Gly, respectively AM8 and AM11 the -OH band is very well defined. With the increase in the content of Gly in the composition, relative to PVA, the intensity of this O-H stretching band increases in the order from AM2 < AM10 ≤ AM9 < AM11 ≤ AM8, and is shifted to the lower wavenumbers, from 3450 to 3358 cm^−1^, strongly suggesting the existence of a polymer hydrogen network formation between glycerol and PVA chains. In fact, this also corresponds to a change in mechanical properties, whereas the plasticizing role of the Gly molecules is evidenced, i.e., destruction of crystalline domain and formation of an amorphous structure of PVA [36]. The main peaks characteristic to PVA, PANi and Gly appear in all materials.

In Figure 2B the FTIR spectra corresponding are organized from top to bottom in the order of increasing PANi. In the 1200–1700 cm^−1^ peaks corresponding to C=C vibration of the quinoid and benzenoid ring of the PANi are found in this region. These two bands are often used to characterize the degree of the oxidation of PANi. Here we note that the emeraldine contains 50% oxidized quinoid component and 50% reduced benzenoid component. We note that the 100% reduced form PANi, (leucoemeraldine), and 100% oxidized form of PANi (pernigraniline) are not conductive [37], but the emeraldine is. The bands attributed to C=C stretching in benzenoid and quinoid rings are found at 1492 cm^−1^ and 1535 cm^−1^. Interestingly, we observe that with the increase in Gly, the relative intensity of the benzoid to quinoid band also increases, for example, in the spectra d–f of Figure 2B, the relative benzoid/quinoid peak intensity ratio increases from 2:1, 4:1 to 8:1 and in the spectra a-c from 1.5:1, 2:1 and 3:1 with increase in Gly weight fraction. Thus, from these data we learn that the conductivity of the PANi may change with the increase in the Gly content in the composite, i.e., becomes more reduced. In other words, it is possible that during the reaction, with the increase in the Gly content the diffusion of the APS oxidant is inhibited by the presence of Gly.

**Figure 3:**
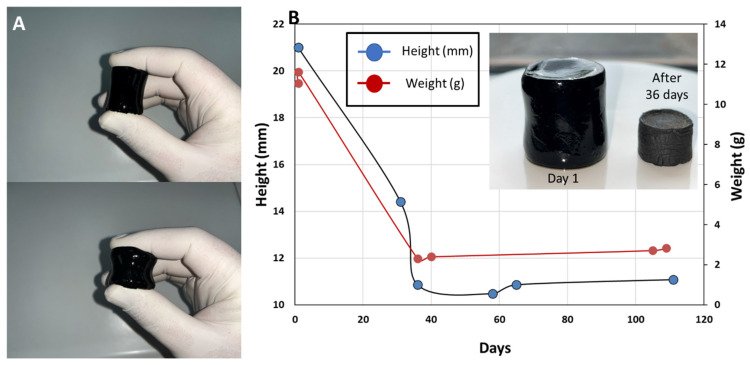
(**A**) Photographs showing the PANi/PVA hydrogel after synthesis, which can be compressed by light squeezing between two fingers. (**B**) Graph depicting the shrinkage in the height of the cylindrical PANi/PVA hydrogel specimen (photograph in the inset) with time, as well as the weight change with time, and its transformation into a rubber-like material after drying at room temperature and ambient conditions.

The FTIR spectra analysis yields major differences between different compositions, as expected, in this work, the only material composition investigated for mechanical and electrical properties is that of PANi/PVA 1:1 and PVA/Gly 1:5. The hydrogel obtained from the latter composition is highly elastic and can be easily compressed by light squeezing between the fingers, Figure 3A, also see Movie 1 in SI. The shape of the material stabilizes after ca. 36 days, as shown in Figure 3B, through the evolution of sample height and sample weight with time. From the curves in Figure 3B it can be clearly seen that the sample height and weight stabilize after 36 days, and the water content is different between the initial weight of the sample from day 1 and the plateau weight after 36 days.

After 36 days of drying at room temperature and ambient conditions, the resistance and the conductivity of the elastic polymer composite were investigated, whereas the electrical measurements were performed with a four-point probe and the measurement method used a current source and a voltmeter, see Figure 1B. The mechanical compression and relaxation cycles were executed with a micromanipulator.

We note that if the polymerization reaction is carried out with the same mixture of reactants, PVA polymer and Gly, before allowing the gelation of the hydrogel, an inhomogeneous mixture was obtained, and the PANi separates out of the reaction. Thus, we emphasize that the key element for obtaining a homogeneous PANi/PVA elastic composite is the formation of a PVA/Gly hydrogel, which acts as a reaction medium. We hypothesize that this slows down the diffusion of the aniline monomers and initiator, through the PVA polymer matrix, slowing down the reaction kinetics and thus producing a homogeneous mixture. The PANi/PVA hydrogel obtained has a gel-like consistency and is highly elastic. Upon drying of the sample, after 36 days at room temperature, the hydrogel matures in a rubber-like material, which can be easily compressed between two fingers; see Movie S1 in Supporting Information (SI).

### 3.2. Influence of the Samples Composition on Electrical Properties

**Figure 4:**
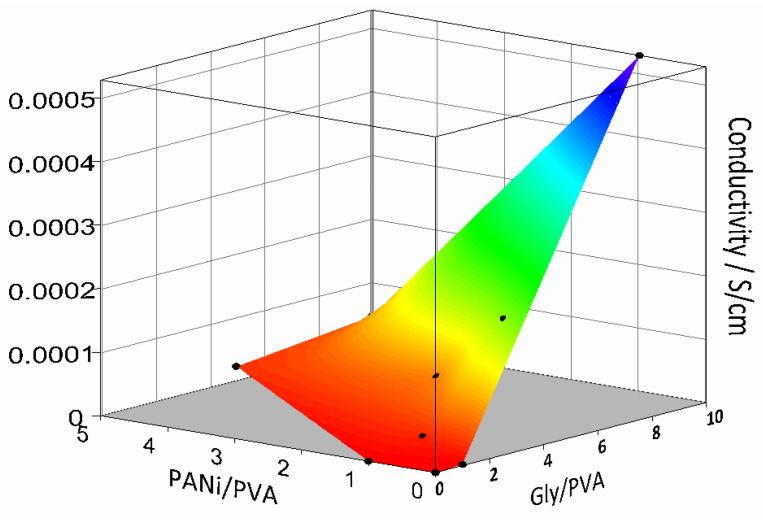
Surface plot showing the conductivity of the elastic PANi/PVA with composition, namely the ratio of the PANi/PVA and Gly/PVA after 60 days drying at room temperature and ambient conditions.

Conductivity of several PANi/PVA composites in the form of films was investigated as a function of their composition after 36 days of drying at room temperature. The surface plot depicted in Figure 4 was constructed from three independent variables, the conductivity of the PANi/PVA composite films as a function of the ratio of the PANi to PVA (PANi/PVA) and PVA to Gly (PVA/Gly). The obtained results are also summarized in Table S1. Several observations can be made. In the absence of PANi and Gly, the PVA material is, as expected, an electrical insulator. With the addition of PANi or Gly in a 1:1 relative proportion to PVA, the conductivity increases, up to 3.6 × 10^−8^ S/cm and 1.7 × 10^−6^ S/cm respectively. Addition of Gly has a stronger effect in increasing conductivity than PANi, see Figure 4. The Gly plays the role of cross-linker and plasticizer, acting as a spacer and increasing the mobility between polymer chains. It can also be observed that the Gly increases the conductivity of the composites. The exact mechanism by which Gly increases the conductivity of the polymer composites is still being debated. Previous studies report that addition of Gly in starch biopolymers plays the role of a plasticizer, improving the mechanical properties of the composite, but also increases its conductivity, up to 4.9 × 10^−5^ S/cm [33]. The authors speculated that, although the composite has been dehydrated, traces of water were responsible for the ionic conductivity but were also involved in ion transport by a Grotthus-type mechanism transferring protons between neighboring sites provided by the glycerol [33]. In a different report, addition of Gly into PEDOT, a well-known conductive polymer, was shown to greatly increase the electrical conductivity of the composite proportional to the fraction of the Gly added [38], also see the Figure 2B. The authors suggested that, due to its plasticizing effect, Gly increased the mobility of the conductive PEDOT chains, causing better interchain contacts and thus enhancing the conductivity of the composite as compared to the PEDOT alone. In the current case both PANi, a conjugated electrically conductive polymer, and Gly contribute to the overall polymer composite conductivity with concurrent conductivity mechanisms, electronic and ionic, respectively. Taking the literature discussion into consideration, it could also be hypothesized that on one hand Gly could play a role in ionic transport and on the other hand the plasticizing could cause an enhanced mobility of the PANi chains and thus better electrical contacts between them, leading to enhanced conductivity.

While the increases in the PANi induces a rubber-like behavior of the composite, the increase in Gly induces softness and flexibility, see the observations in Table 1 and Table S1. With an increase in both PANi and Gly in the composition, the material conductivity increases to a maximum of 5.3 × 10^−4^ S/cm for PVA/Gly 1:10 and PANi/PVA 1:1 and 1.7 × 10^−4^ S/cm for PVA/Gly 1:5 and PANi/PVA 1:1. While the former composite has a gel-like, very soft and sticky composition, the latter has a rubbery characteristic. Due to the better mechanical properties of the former, we chose for further investigation in this work the PANi/PVA composite with the following composition PVA/Gly 1:5 and PANi/PVA 1:1.

### 3.3. Mechanical Properties

**Figure 5:**
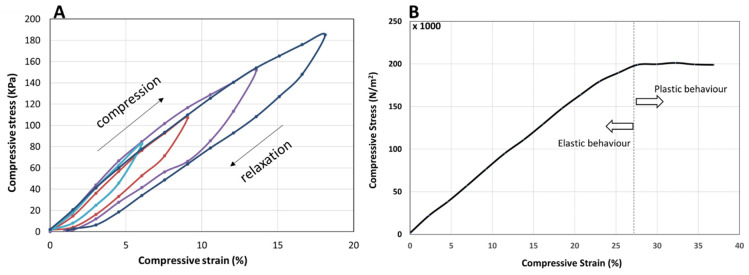
(**A**) Compression (loading)—relaxation (unloading) cycles depicted as stress-strain curves, showing viscoelastic behavior, reflected in the hysteresis, which is increasing with the increase in the maximum load. The compression-relaxation cycles (curves) differing in their maximum load are depicted in different colors. (**B**) Stress vs. strain curve of the PANi/PVA composite in compression mode. The linear portion of the curve represents the elastic behavior of the material, while the plastic behavior begins beyond a ~27% longitudinal compression of the cylindrically shaped PANi/PVA composite.

The mechanical properties of the PANi/PVA material, obtained after ca. 36 days drying at room temperature, were determined by applying an incremental load (stress) and measuring the longitudinal deformation (strain), in compression mode, as depicted in Figure 5. The PANi/PVA composite exhibits a viscoelastic behavior reflected in the increase in the hysteresis loop upon loading (compression) and unloading (relaxation) cycles, as depicted in Figure 5A. While for purely elastic materials the loading and unloading curves are superimposed, the viscoelastic materials exhibit an increasing hysteresis loop with the increase in the applied load, which is visible in the curves of Figure 5A. Further, the area enclosed by the loop represents the energy loss dissipated as heat, which indicates that this composite can be used as shock absorber. Under longitudinal compression the material exhibits an elastic behavior up to a 27% compression, after which it exhibits a plastic behavior, see Figure 5B. The Young’s modulus calculated from the slope of the linear portion of the stress vs. strain curve is 8.4 × 10^−4^ GPa; this value places the material in the category of rubber-like materials [39]. We note that the elastic properties of the composite can change as a function of drying time. For example, different sample shapes and sizes, may have a different rate of drying, thus the elastic properties should be measured after allowing the sample to fully mature from the hydrogel into the rubbery material in the final stages.

### 3.4. Change of Electrical Properties with Mechanical Compression and Relaxation

To monitor changes in electrical properties, resistance of a cylindrical sample with a diameter of 6.8 mm and height of 16.56 mm, was compressed on its long axis between two moving Cu electrodes controlled by a micromanipulator (0.01 mm precision). During compression the resistance of the sample was monitored.

**Figure 6:**
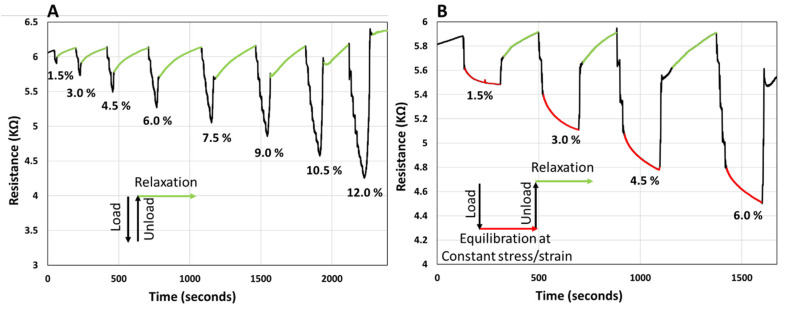
(**A**) Graph showing the electrical resistance of PANi/PVA composite with load (black)-unload (black)-relaxation cycles (green) cycles. The incremental load is expressed by the compressive strain (%). (**B**) Graph showing the electrical resistance of PANi/PVA composite with load (black)-equilibration at constant stress/strain (red)-unload (black)-relaxation (green) cycles. Throughout the equilibration stage (the red curve) the compressive strain is constant. For both graphs, throughout the relaxation stage (the green curve) the applied compressive strain is 0%. The incremental load is expressed by the compressive strain (%). All cycles were applied in the elastic regime of the sample.

To observe the resistance response, the sample was compressed by applying an incremental load in steps of 0.7 N with 10 s waiting time before each increment. With the application of the load the resistance of the sample decreases. First, load-unload-relaxation cycles were applied, as depicted in Figure 6A. In this case, after the maximum load was applied, the sample was quickly unloaded, and the resistance was monitored until it reached the initial baseline. From the data presented in Figure 6A, it can be observed that the resistance recovers to the initial baseline after some time; portions of the curve in Figure 6A, corresponding to the relaxation of the sample after unloading are coded in green. The recovery process appears to be slow, and the recovery time appears to be somewhat proportional to the load applied, at least at small strains, i.e., <9%.

Next, the resistance of the sample was monitored during the application of load-equilibration at constant stress/strain-unload-relaxation cycles, Figure 6B. In Figure 6B the portions of constant stress are coded red and regions corresponding to relaxation after unloading are coded green. For the red regions, when maximum load was reached, at constant stress/strain, the resistance appears to further decrease, exponentially. After 3 min under constant stress/strain, the sample was unloaded, and the relaxation was allowed until the resistance recovered to the baseline (green regions). The recovery time of the resistance during the relaxation stage appears to be proportional to the load applied. It can be noted that monitoring the electrical resistance, a bulk parameter, reflects the intrinsic phenomena and transformation that take place within the sample. The fact that the resistance of the sample is at non-equilibrium under stress (load) and recovers at timescales on the order of minutes after removing the stress appears to be justified in part by the viscoelastic mechanical response of the sample, as observed in Figure 5A. Therefore, it is justified to assume that the electrical resistance is an accurate reflection of the internal strain and shape transformations in the sample. In fact, monitoring any shape changes in the sample with the electrical resistance of a viscoelastic material could be a very valuable way to monitor tactile pressure, a technology that can be expanded to interfacing human motion and development of intelligent prosthetics, mattresses, cushions, and other comfort devices. Development of artificial skin for robots, to interpret the tactile interactions with their environment could also be a potential application for viscoelastic composites, such as PANi/PVA composite studied here. As a further practical application this memory effect can be used to track the history of the material usage and could provide important information on the previous interaction of the material with the environment, as a way of basic information storage.

### 3.5. Non-Linear Viscoelastic Response

To better understand how resistance can be connected to monitoring the internal transformations within the PANi/PVA composite, we can assume that the resistance is proportional to the internal strain in the sample. One such model is the Kohlrausch–Williams–Watts function (KWW) [40,41,42], which is a stretched exponential correlation function:

(1) $$K\left(t\right)=K_{0}exp\left(-{\left(\frac{t}{\tau }\right)}^{\beta }\right)$$

where *K*(*t*) is the relaxing quantity (in this case the electrical resistance), *t*—relaxation time, *τ*—is the relaxation time at which the *K*(*t*) decays to the value of 1/e and is related to the damping properties of the material (when *τ* decreases, better shock absorption properties are obtained). 0 < *β* < 1 is the breath of the relaxation time distribution and is based on the assumption that every degree of freedom, e.g., for the polymer chains in a matrix, relaxes independently with a characteristic time *τ_i_* [43].

**Figure 7:**
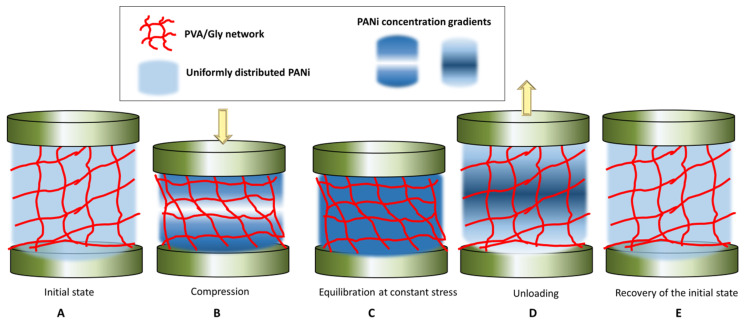
Depiction of the hypothetical relaxation mechanism of the PVA network and of the PANi polymer during compression-equilibration-decompression-relaxation cycles.

The KWW function has been recognized as the appropriate model for describing the non-linear viscoelastic behavior of the materials, in contrast to the Maxwell model that describes the behavior of viscoelastic materials as a linear combination of Hookean springs and Newtonian dashpots, typically modeled by the simple Debye decaying exponential function [43]. In the KWW equation, for *β* = 1, the KWW becomes the standard Debye function used to model relaxation of viscoelastic materials [42,43]. While the physical meaning of *β* is still being debated, the *β* term can be related to the coupling parameter *n* = 1 − *β*, which is degree of intermolecular co-operativity and degree of molecular coupling within the polymer [40]. KWW relates the relaxation time *τ* and the molecular behavior of the material, reflected in the value of *n*. For a small *n* and a large *β*, the polymer segments are less intermolecularly constrained, while vice versa for a large *n* and a small *β*, the interaction amongst neighboring segments builds up to a degree that retard the relaxation [43]. By fitting the KWW function, the Equation (1), where *K*(*t*) is replaced by resistance *R*(*t*), to the red and green regions of the experimental data in the curve of Figure 6B, we obtain the values of *τ*, *β* and *n*, given in Table 2. The fitted curves are given in Figure S4. From the obtained data, it can be observed that the values of *β* are smaller than unity, suggesting that the material has indeed a non-linear viscoelastic behavior. Further, *β* oscillates between 0.7 to 0.9, which suggests a varying degree of intermolecular constraint with the level of compression. For example, at constant stress, with the increase in compressive strain, the value of *β* decreases, *η* increases, which retards the relaxation time, and *τ* increases. In other words, with increase in the maximum stress applied, the relaxation of the material becomes increasingly hindered, i.e., the PANi, PVA chains and Gly components of the composites become increasingly intermolecularly constrained. The lowering of the resistance with sample compression suggests that the conductive PANi chains come closer together, thus increasing the conductivity of the sample. The equilibrium value of the resistance is, however, not achieved, at constant stress/strain, but is slowly decreasing as shown in the red regions of the curve in Figure 6B. We hypothesize that this is due to two processes that take place. Firstly, during compression (loading) there is a local gradient in concentration of the conductive PANi building up near the compressing electrodes, Figure 7B. Secondly, when the maximum load is reached, and stress is kept constant, the PANi concentration gradient is dissipated by slow diffusion and redistribution of the conductive PANi polymer in the sample, Figure 7C. It is reasonable to assume that, when maximum stress is increased, this diffusion and redistribution of PANi becomes slightly hindered, possibly due to enhanced polymer interchain interaction or decrease in the polymer pore network size. This hypothesis is supported by the trend in the values of *τ*, *β* and *η* during relaxation, after removing the load. After removal of the load the sample recovers its shape, while the sample resistance takes significant time to recover. We believe the dissipation of PANi concentration gradients, built up during the constant stress, Figure 7C, is also responsible for the observed slow recovery of the resistance, Figure 7D,E. From Table 2, during relaxation after removing of the load, *τ* remains roughly independent of the maximum load applied but *β* increase to 0.9 and *η* decrease to 0.1, after the sample has been hardest compressed, which suggests that indeed the building up of conductive PANi concentration gradients in the sample and diffusion could be responsible for the variation in the sample conductivity with compression and relaxation. This behavior, of lag in resistance recovery at equilibration and relaxation stages observed for the PANi/PVA composite obtained after the drying of the hydrogel is not seen for freshly prepared hydrogels that are imbibed with water (see the PPy/PVA hydrogel electrical resistance with compression in Figure 6d reported by Lu et al. [31]). The reason for this is that in a freshly prepared hydrogel the main electron transport mechanism is through the water medium, whereas the charge carriers are ions, while for the dried composite the intrinsic conductivity of the conductive polymer must be contributing at least in part to the observed effect.

### 3.6. Memory of Deformation in the Plastic Regime

**Figure 8:**
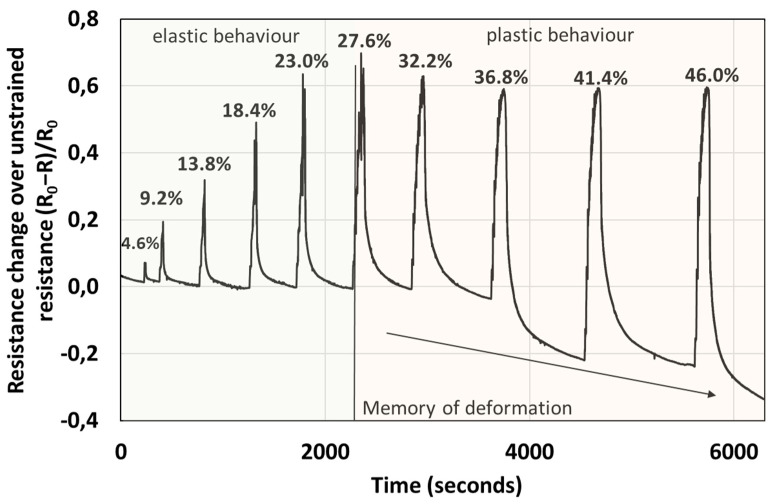
Graph depicting the resistance change over unstrained resistance with compression. There are two different regimes, the elastic regime, where the resistance change is proportional to the applied force, and the plastic regime where the resistance plateaus. The labels of the peaks correspond to compressive strain of the material. In the plastic regime, strain > 27%, the recovery of the resistance in the relaxation stage increases beyond the baseline, proportionally to the force applied in the plastic regime. Note that the applied strain in the relaxation stage is 0%. This observed effect we call the memory of plastic deformation.

The electrical response, i.e., change in resistance, is proportional to the force applied in the elastic regime, see Figure 6 and Figure 8. Interestingly, the percentage change in resistance over the unstrained resistance (R_0_ − R/R_0_) is almost within a factor of 2 larger than the percentage compressive strain. For example, for a 23% strain the resistance change of the material is almost 60%, in the elastic regime. However, when the sample is pressed, such that plastic deformation takes place, the resistance of the sample does not increase proportionally to the force applied, but plateaus. Further, during the relaxation stage, the resistance does not recover to the initial values, but resistance appears to increase with the level of plastic deformation (the change in resistance to unconstrained resistance falls below the baseline into the negative values), a phenomenon which we call “memory of plastic deformation”. For example, this situation is depicted in Figure 8, where in the elastic regime, the maximum of the R/R_0_ peaks of the material is proportional to the force applied and deformation (longitudinal strain). After the decompression and relaxation stage the R/R_0_ decreases (resistance increases), recovering to the initial baseline. However, in the plastic regime the increase in R/R_0_ is not proportional with the maximum compression force and deformation (longitudinal strain). In fact, it appears that the maxima of R/R_0_ peaks decrease and reach a plateau. Interestingly, in this regime after decompression and during the relaxation stage, the R/R_0_ does not recover to the original baseline, but the baseline also decreases, apparently proportional to the force applied. While the apparent shape of the material is preserved, the memory of deformation is gauged in terms of R/R_0_ of material rather than its apparent shape. Thus, we consider that such materials could be useful in designing new prosthetics that enable live monitoring of the pressure, stress and strain of the material via electrical signals.

## 4. Conclusions

In this work, we have shown that a PANi/PVA flexible composite, consisting of a semiconductive PANi polymer, an electrically insulating PVA polymer and glycerol, which acts as a plasticizer and cross-linker, can be prepared by in situ polymerization of PANi in hydrogel matrix at low temperatures. The key towards obtaining a homogeneous PANi/PVA composite is that the semiconducting PANi forms in the hydrogel matrix. We believe that when aniline monomer is polymerized in the hydrogel matrix the polymerization reaction is diffusion controlled, which slows down the reaction and a homogeneous composite is obtained. Immediately after synthesis, the material is a highly elastic hydrogel and after drying at room temperature in ambient conditions it matures into a rubber-like material. The PANi/PVA rubber obtained is conductive with damping and shock-absorbing properties, i.e., viscoelastic properties. Upon application of a mechanical stress, e.g., compression, the resistance of the material decreases significantly, by at least one order of magnitude. We have demonstrated that in the elastic regime the change in resistance is directly proportional to the applied stress. Such properties can make it useful in developing artificial skins for humanoid robots, to interpret the tactile pressure as an electrical signal for an improved interaction with their environment. Further, when the mechanical stress applied to the PANi/PVA composite exceeds the elastic regime, in the plastic regime, the resistance of the material does not recover when the load is removed, thus exhibiting a memory effect. This phenomenon can be used to develop new prosthetics to monitor discomfort areas and high-pressure points. Further, we have proposed a new way to monitor the relaxation of the viscoelastic PANi/PVA composite by modelling the relaxation of the resistance at constant stress/strain with the KWW function. While the apparent shape of the material at constant loads and in the absence of loads does not change, changes in resistance following the skewed KWW exponential function in time indicate that intrinsic transformation within the material at the molecular level take significant time to equilibrate. Further work should be focused on optimizing the synthetic conditions and using this material in real-life applications. Further, it should be noted that before employing this material in real-life applications it should be engineered such that maturation conditions, diffusion of components, time of measurements, and the stability of electrical and mechanical signals with measurement cycles should be exhaustively determined.

## Figures and Tables

**Table 1:** Composition of the hydrogel according to the weight ratio of the components PVA:Gly, PVA:Gly and the molar ratio between the aniline monomer (ANi) to the APS oxidant initiator.

Sample Name	PVA:Gly (Weight Ratio)	PVA:Gly (Weight Ratio)	ANi:APS (Molar Ratio)	Observations
AM1	1:0	1:1	1:1	hard rubber
AM2	1:2	1:1	1:1	rubbery
AM3	1:0	1:0	-	glassy, hard, brittle
AM4	2:1	1:0	-	soft, sticky
AM7	1:5	1:1	1:1	rubbery
AM8	1:10	1:1	1:1	soft, gel-like
AM9	1:5	1:2	1:1	brittle rubber
AM10	1:5	1:5	1:1	brittle rubber
AM11	1:10	1:5	1:1	brittle rubber

**Table 2:** The values for *τ*, *β* and *n* obtained by fitting the Equation (1) to the portion of the relaxation at constant strain.

**Compressive Strain** **%**	***t*** **(s)**	**b**	***n***
1.5	47.0	0.8	0.2
3.0	107.6	0.8	0.2
4.5	197.4	0.7	0.3
6.0	322.2	0.7	0.3
**Relaxation after** **the maximum** **compressive strain %**	***t*** **(s)**	**b**	***n***
1.5	775.7	0.7	0.3
3.0	912.2	0.8	0.2
4.5	857.0	0.9	0.1

## Data Availability

All data is available upon request from the corresponding author.

## Supplementary Materials

The following supporting information can be downloaded at: https://www.mdpi.com/article/10.3390/polym14214638/s1, Table S1. Conductivity function of composition, relative weight content of PANi and Glycerol to PVA; Figure S1. Photograph of the electrical measurement set-up: (A) the four-point probe set-up with disk shaped copper electrodes, whereas the four-point contacts were achieved by using hemispherical copper disks glued together by electrically insulating epoxy resin; (B) the PANi/PVA composite fixed between the copper electrodes; (C) cylindrical PANi/PVA composite immobilized between the copper electrode, the top Cu electrode is controlled by a micromanipulator; Figure S2. SEM images of the (A) AM1, (B) AM3, (C) AM4, and (D) AM7; Figure S3. FTIR spectra of (a) PVA, (b) Gly and (c) PANi; Figure S4. Fitting of the equilibration (A) and relaxation regions (B) of the curve in Figure 4B, main text, with the Kohlrausch-Williams-Watts function (KWW) function, Equation (1). The experimental data is represented by the dotted line and the fits by the solid lines. For better visualization, the curves in graph (A) were offset on the vertical axis by the quantities shown.
