# Supplementary material for: Flexible Composites with Variable Conductivity and Memory of Deformation Obtained by Polymerization of Polyaniline in PVA Hydrogel
Source: Polymers (Basel). 2022 Oct 31;14(21):4638. doi: 10.3390/polym14214638 (PMC9657478; doi:10.3390/polym14214638)
Supplement: Supplementary file 1 [file polymers-14-04638-s001.zip › 2022.10.21_SI_PANi Hydrogel _AH_final AS.pdf]

Supporting Information

Flexible Composites with Variable Conductivity and Memory of Deformation Obtained by Polymerization of Polyaniline in PVA Hydrogel

Andrei Honciuc[1]*, Ana-Maria Solonaru[1], Mirela Teodorescu[2]

"Petru Poni" Institute of Macromolecular Chemistry, Aleea Gr. Ghica Voda 41A, Iasi, 700487, Romania

[1]Electroactive Polymers and Plasmochemistry Laboratory

[2]Laboratory of Natural Polymers, Bioactive and Biocompatible Materials

*Corresponding email: honciuc.andrei@icmpp.ro

Table S1. Conductivity function of composition, relative weight content of PANi and Glycerol to PVA, whereas the PVA reference concentration is unity.

Sample name	Conductivity (S/cm)	PANi:PVA	Gly:PVA	Observations
AM2	1.6E-05	1: 1	2: 1	rubbery
AM7	1.7E-04	1: 1	5: 1	rubbery
AM8	5.3E-04	1: 1	10: 1	soft, gel-like
AM9	6.0E-05	2: 1	5: 1	brittle rubber
AM10	2.2E-05	5: 1	5: 1	brittle rubber
AM11	4.6E-05	5: 1	10: 1	brittle rubber
AM1	3.6E-08	1: 1	0: 1	hard rubber
AM3	1.0E-10	0: 1	0: 1	glassy, hard, brittle
AM4	1.7E-06	0: 1	0.5: 1	soft, sticky

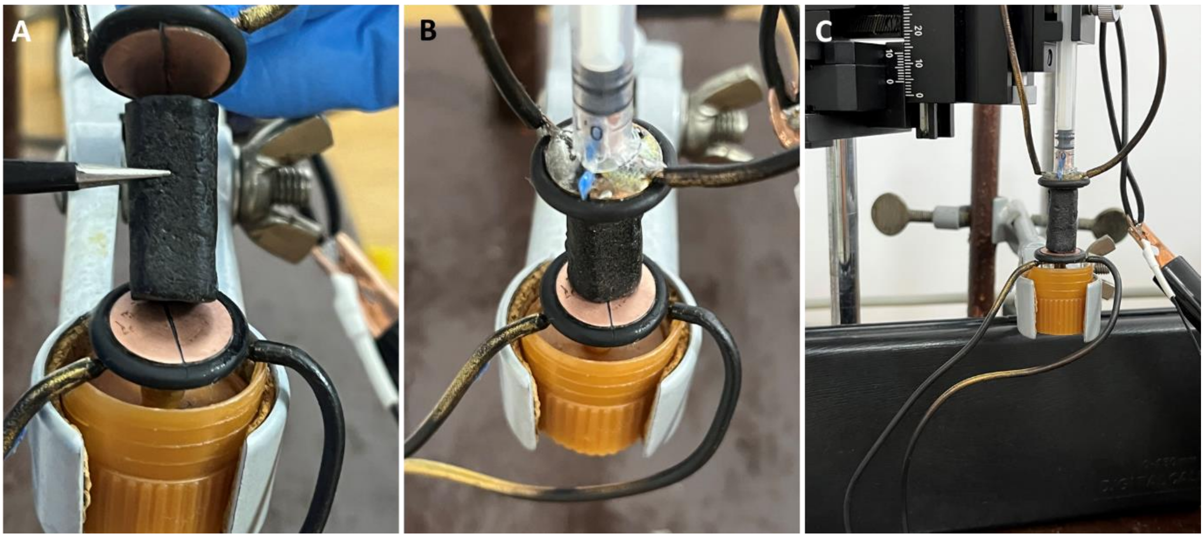

Figures S1. Photograph of the electrical measurement set-up: **(A)** the four-point probe set-up with disk shaped copper electrodes, whereas the four-point contacts were achieved by using hemispherical copper disks glued together by electrically insulating epoxy resin; **(B)** the PANi/PVA composite fixed between the copper electrodes; **(C)** cylindrical PANi/PVA composite immobilized between the copper electrode, the top Cu electrode is controlled by a micromanipulator.

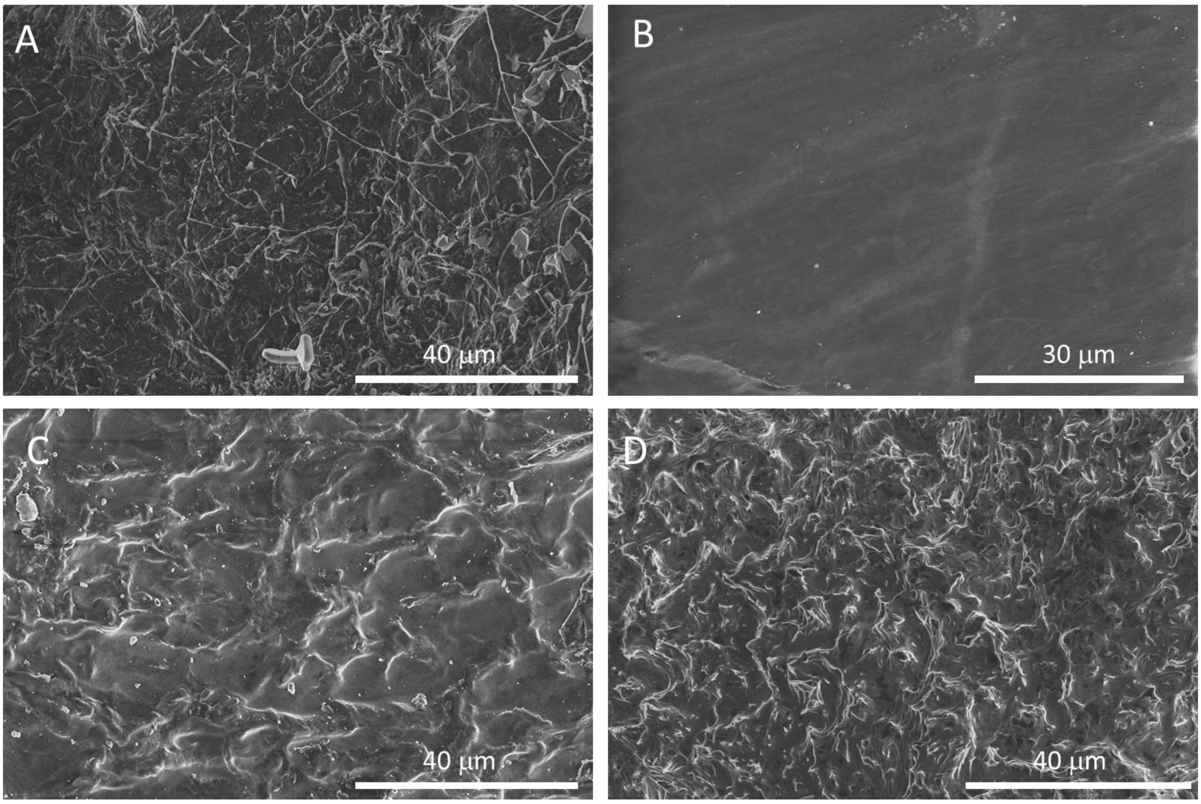

Figure S2. SEM images of the (A) AM1, (B) AM3, (C) AM4, and (D) AM7.

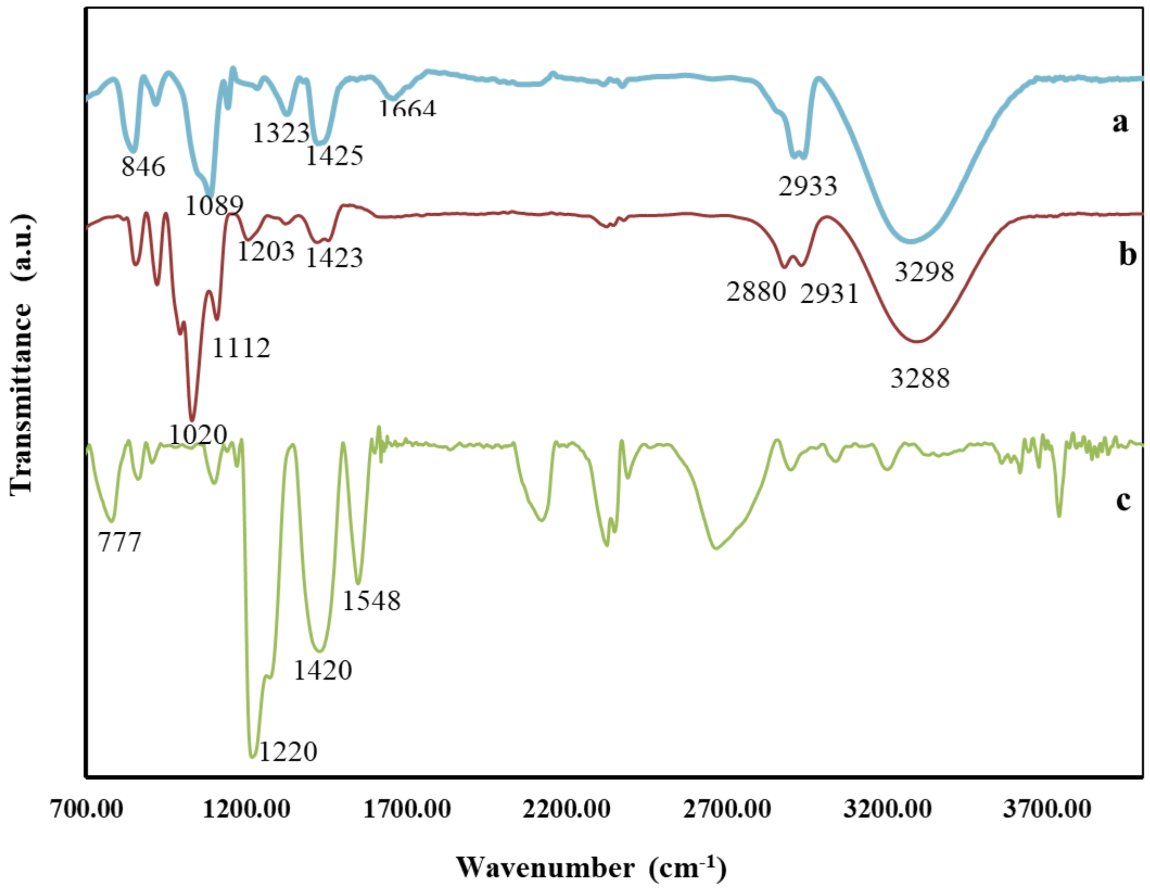

Figure S3. FTIR spectra of a) PVA, b) Gly and c) PANi.

Figure S3 shows the FTIR spectra of pure components PANi, Gly and PVA used in the synthesis of the composite. First, in the spectrum of PVA the characteristic bands appear at 3298 cm^{-1} that can be assigned to the O-H stretching vibration, while that at 2933 cm^{-1} and 1425 cm^{-1} are specific to stretching vibration and bending vibration of C-H in CH_2. At 1664 cm^{-1} we find the band specific to the stretching of C=O group, most likely originating in the acetate impurities (PVA is obtained from acetate by hydrolysis)[1].

Gly spectrum presents a wide band at 3288 cm^{-1} which is due to O-H group. The bands at 2931 cm^{-1} and 2880 cm^{-1} belong to the C-H. stretching of the alkyl groups [2]. The peaks at 1423 cm^{-1} and 1215 cm^{-1} correspond to the C-H bonds and those from 1020 cm^{-1} and 1112 cm^{-1} is attributed to C-O bonds [3]

PANi spectrum presents the main bands located at 1548 cm^{-1} assigned to C=C stretching of the quinoid rings, at 1420 cm^{-1} due to C=C stretching vibration of benzenoid rings, at 1220 cm^{-1} –C-N stretching vibration and at 777 cm^{-1} C-H out of plane bending of 1,4 rings [4]. In the region 2250 -2700 cm^{-1} in FTIR spectrum appear the ammonium ions (NH_4^+)

and because the synthesis of polyaniline was performed using APS as oxidant the peaks specific to ammonium cations, which inevitably remain in the sample, can also appear in this region.

.

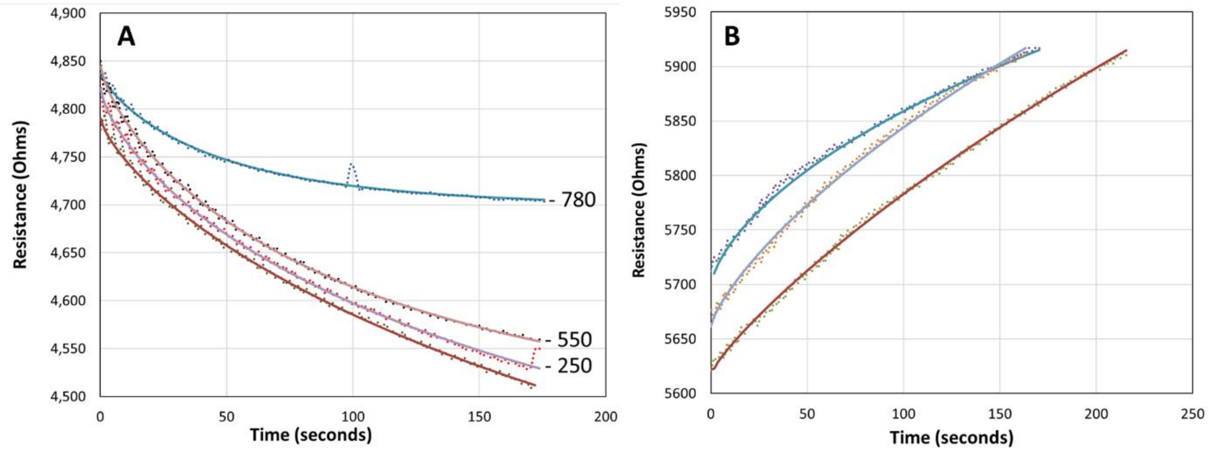

Figure S4. Fitting of the equilibration **(A)** and relaxation regions **(B)** of the curve in Figure 4B, main text, with the Kohlrausch-Williams-Watts function (KWW) function, Equation 1. The experimental data is represented by the dotted line and the fits by the solid lines. For better visualization, the curves in graph (A) were offset on the vertical axis by the quantities shown.

References

1. Brza, M.A.; Aziz, S.B.; Anuar, H.; Ali, F. Structural, Ion Transport Parameter and Electrochemical Properties of Plasticized Polymer Composite Electrolyte Based on PVA: A Novel Approach to Fabricate High Performance EDLC Devices. *Polymer Testing* **2020**, *91*, 106813, doi:10.1016/j.polymertesting.2020.106813.
2. Mohsin, M.; Hossin, A.; Haik, Y. Thermal and Mechanical Properties of Poly(Vinyl Alcohol) Plasticized with Glycerol. *J. Appl. Polym. Sci.* **2011**, *122*, 3102–3109, doi:10.1002/app.34229.
3. Oliveira, A.C.S.; Santos, T.A.; Ugucioni, J.C.; Rocha, R.A.; Borges, S.V. Effect of Glycerol on Electrical Conducting of Chitosan/Polyaniline Blends. *J Appl Polym Sci* **2021**, *138*, 51249, doi:10.1002/app.51249.

4. Solonaru, A.-M.; Asandulesa, M.; Honciuc, A. Homologous Series of Polyaniline Derivatives Block Copolymers with Amphiphilic and Semiconducting Properties. *Polymers* **2022**, *14*, 2149, doi:10.3390/polym14112149.
